# Enhanced calcium carbonate-biofilm complex formation by alkali-generating *Lysinibacillus boronitolerans* YS11 and alkaliphilic *Bacillus* sp. AK13

**DOI:** 10.1186/s13568-019-0773-x

**Published:** 2019-04-11

**Authors:** Yun Suk Lee, Woojun Park

**Affiliations:** Laboratory of Molecular Environmental Microbiology, Department of Environmental Science and Ecological Engineering, Korea University, Seoul, 02841 Republic of Korea

**Keywords:** Alkaline generation, Dual species CaCO_3_ precipitation, Bacteria-CaCO_3_ interaction, Branched chain fatty acid synthesis, Membrane rigidity

## Abstract

Microbially induced calcium carbonate (CaCO_3_) precipitation (MICP) is a process where microbes induce condition favorable for CaCO_3_ formation through metabolic activities by increasing the pH or carbonate ions when calcium is near. The molecular and ecological basis of CaCO_3_ precipitating (CCP) bacteria has been poorly illuminated. Here, we showed that increased pH levels by deamination of amino acids is a driving force toward MICP using alkalitolerant *Lysinibacillus boronitolerans* YS11 as a model species of non-ureolytic CCP bacteria. This alkaline generation also facilitates the growth of neighboring alkaliphilic *Bacillus* sp. AK13, which could alter characteristics of MICP by changing the size and shape of CaCO_3_ minerals. Furthermore, we showed CaCO_3_ that precipitates earlier in an experiment modifies membrane rigidity of YS11 strain via upregulation of branched chain fatty acid synthesis. This work closely examines MICP conditions by deamination and the effect of MICP on cell membrane rigidity and crystal formation for the first time.

**Electronic supplementary material:**

The online version of this article (10.1186/s13568-019-0773-x) contains supplementary material, which is available to authorized users.

## Introduction

Calcium carbonate precipitating (CCP) bacteria contribute to the geochemical cycle as they precipitate carbonate minerals, including calcium carbonate in nature (Douglas and Beveridge 1998). Formation of stromatolite is an example of calcium carbonate precipitation by cyanobacterial-bacterial mat communities (Paerl et al. 2001). There are four main environmental parameters that govern reaction kinetics for calcium carbonate precipitation: (1) pH, (2) calcium (Ca^2+^) ion concentration, (3) dissolved inorganic carbon (DIC) concentration, and [4] nucleation site availability (Hammes and Verstraete 2002). Bacteria influence these parameters through their metabolic activity, the production of biofilm, and exopolysaccharide (EPS) formation, eventually leading to microbially induced CaCO_3_ precipitation (MICP). Bacterial metabolic pathways can create compounds that increase the solution pH. These include photosynthesis, ureolysis, ammonification, denitrification, sulphate reduction, and formate oxidation are known to induce calcium carbonate precipitation (Hammes and Verstraete 2002; Ganendra et al. 2014). When resultant pH and DIC increases are accompanied by the aforementioned pathways in an environment with calcium ions, bacterial cell walls or their extracellular polymeric substances (EPSs) can serve as a nucleation site for precipitation reaction (Hammes and Verstraete 2002). Despite the fact that alkaline generating bacteria are ubiquitous in the natural environment, much of their microbial ecology remains to be investigated.

Bacteria can form biofilms in most environmental niches, and nearly all biofilm communities in nature comprise a variety of species (Elias and Banin 2012). Biofilms provide protection to cells when bacteria face fluctuating and harsh environmental conditions. They facilitate complex interactions between individual cells and an environment (Hall-Stoodley et al. 2004; Liu et al. 2016). The biofilm matrix is composed of multiple species and a mixture of components such as EPS, protein, nucleic acids, and other substances. The best studied one of these components is EPS (Davey and O’Toole 2000). The co-occurrence pattern of bacteria embedded in this matrix is determined by species interaction (Nadell et al. 2016). Biofilms not only contain biogenic substances, but also contain granules trapped from sediments (Riding 2000). EPSs generally possess metal binding properties, including Ca^2+^, because EPSs retain high molecular weight compounds that have charged functional groups (Bhaskar and Bhosle 2006). Therefore, multi-species biofilm in nature can act as a foundation for MICP. Previous studies have shown that EPS formed by biofilms is crucial when investigating bacterial physiological functions and activities (Giuffre et al. 2013; Braissant et al. 2003). These ex situ research efforts have also found that EPS could regulate spatial position of precipitation during mineralization. In the absence of microscale chemical gradients, nucleation models have estimated that crystals produced by biofilms can distribute randomly in EPS (Arp et al. 2001). Most current studies of biofilms and biomineralization suggest that precipitation of mineral appears on the biofilm surface primarily (Zhang and Klapper 2010). However, these studies and models are not suitable for in situ observation since information of mineral formation in biofilms, such as spatial patterns, are lacking. A recent study has developed a method for a real time, in situ biomineralization examination by imaging the biomineralized calcium carbonate within biofilms produced by *Pseudomonas aeruginosa* (Bai et al. 2017). However, interdisciplinary research studies of multi-species embedded biofilm, and its resultant MICP, have not been reported yet. Exploring the relationship between pH increasing bacteria involved in multispecies biofilm and calcium carbonate formation is important for understanding integral mechanisms of microbially induced calcium carbonate formation (MICP).

All living organisms, including bacteria, require metal elements for metabolic activities as metals have structural or catalytic roles (Ehrlich 1997). Among many metal ions, iron (Fe^2+^), magnesium (Mg^2+^), manganese (Mn^2+^), zinc (Zn^2+^), copper (Cu^2+^), and Ca^2+^ are reported to possess important cellular functions in microorganisms (Roane et al. 2009). Various functions of metals, including Fe^2+^, Mg^2+^, Zn^2+^, and Mn^2+^, in bacterial cells have been well scrutinized (Andrews et al. 2003; Diaz-Ochoa et al. 2014). However, the role of Ca^2+^ inside bacteria remains far from clear (Norris et al. 1996; Dominguez 2004). Functions of Ca^2+^ in eukaryotes are well-established compared to those in prokaryotic cells. Ca^2+^ can act as a signaling molecule and a secondary messenger that is tightly regulated by the gradient between intracellular and extracellular Ca^2+^ concentrations (Islam 2012). It has been recently found that Ca^2+^ also possesses signaling functions in prokaryotic cells, including bacteria. Ca^2+^ concentration inside cell is tightly controlled. It is much lower in concentration than that in the outside environment (Dominguez 2004; Islam 2012; Dominguez et al. 2015). Furthermore, Ca^2+^-binding proteins pertaining EF-hand motifs are found to be distributed among various bacterial species (Dominguez et al. 2015; Zhou et al. 2006; Rigden et al. 2011). Recent studies suggest that the delicate Ca^2+^ signaling found in eukaryotes might have evolved from prokaryotic signaling (Shemarova and Nesterov 2005; Case et al. 2007). Recent studies are unveiling specific roles of calcium in bacteria, such as Ca^2+^-signaling, Ca^2+^-binding proteins, motility, spore germination, quorum sensing, EPS production, and biofilm formation (Bertrand et al. 2010; Bilecen and Yildiz 2009; Cruz et al. 2014; Guragain et al. 2013; Johnson et al. 2011; Porsch et al. 2013; Sarkisova et al. 2005). It has been found that Ca^2+^-transporting ATPases, motility proteins, and two-component systems in bacteria can act in calcium concentration dependent ways (Bertrand et al. 2010; Bilecen and Yildiz 2009; Cruz et al. 2014; Guragain et al. 2013; Johnson et al. 2011; Porsch et al. 2013; Sarkisova et al. 2005). Because MICP occurs when Ca^2+^ exists in surrounding, CCP bacteria can provide a physiological model to examine bacterial response to Ca^2+^ when they produce calcium carbonate minerals.

In our previous study, a novel CCP bacterium *Lysinibacillus* sp. YS11 was isolated and characterized for its ability to produce calcium carbonate in solution (Lee et al. 2017). Unlike increase in pH level via ureolysis which has been broadly studied regarding MICP, strain YS11 was found to utilize pathways other than urea hydrolyses for pH increase of a solution (Yan et al. 2017). Here, we found that alkaline generation of YS11 was through ammonia production via deaminase activity. Alkaline generation from YS11 facilitated the growth of alkaliphilic bacterium *Bacillus* sp. AK13, which was isolated from the identical environment. It altered the morphology and amount of biofilm-bound calcium carbonate. Furthermore, we demonstrate that the precipitated calcium carbonate can modify membrane rigidity in bacteria by upregulating branched chain amino acid (BCAA) and branched chain fatty acid (BCFA) synthesis.

## Materials and methods

### Genomic DNA extraction, whole genome sequencing, and genomic analysis of YS11

Genomic DNA of *Lysinibacillus boronitolerans* YS11 was extracted using Wizard Genomic DNA purification Kit (Promega, USA). Whole-genome sequencing was performed using Pac Bio RSII Single Molecule Real Time (SMRT) sequencing method (Pacific Biosciences, USA) with a SMRTbell template library in Chunlab (Seoul, South Korea) according to manufacturers’ instructions. The complete genome was achieved using CLgenomics program provided by Chunlab. Gene annotation and assembly were conducted with NCBI Prokaryotic Genomes Automatic Annotation Pipeline. CLgenomics program provided by Chunlab and kegg pathway searching were used for genomic analysis of YS11.

### *L. boronitolerans* YS11 culture conditions

For growth test of strain YS11, a seed culture was incubated in Luria-Bertani (LB) medium, which is composed of 1% tryptone (Bioshop), 1% sodium chloride (Sigma-Aldrich), .5% yeast extract (Bioshop), at 30 °C overnight with shaking at 220 rpm. Next, 1 ml of the seed culture was transferred to a 1.7-ml microtube and washed twice with phosphate buffered saline (PBS), which is composed of .08% NaCl, .02% KCl, .144% Na_2_HPO_4_, .024% KH_2_PO_4_. Next, strain YS11 at density of 1 × 10^6^ CFU/ml was transferred into 25 ml of calcium acetate (CaAc) medium (15.8 mM calcium acetate, .4% yeast extract, .5% glucose) or sodium acetate (NaAc) medium (15.8 mM sodium acetate, .4% yeast extract, .5% glucose) in a 50-ml flask to observe the growth on Ca-rich or Ca-poor condition. Growth curves were generated based on colony-forming unit (CFU). The culture was serially diluted from 10^0^ to 10^−8^ using PBS for dilution. Then, 200 µl of the diluted bacteria was streaked into pre-heated LB plates. The colonies were counted after 12 h incubation at 30 °C.

### RNA isolation and transcriptomic analysis by RNA-seq

Total RNA was obtained from mid-exponentially grown YS11 cells (6 h) from NaAc and CaAc media using a RNeasy kit (Qiagen, Hilden, Germany) following the manufacturer’s instructions. The extracted total RNA was then sent to Chunlab (Seoul, South Korea) for RNA sequencing and alignment. Ribo‐Zero rRNA removal kit (Epicentre, Medison, WI, USA) was used for ribosomal RNA depletion according to the manufacturer’s instructions. Libraries for Illumina sequencing were constructed with TruSeq Stranded mRNA sample prep kit (Illumina, San Diego, CA, USA) following the manufacturer’s protocol. RNA sequencing was conducted on Illumina HiSeq 2500 platform using single-end 50 bp sequencing. Sequence data for the reference genome (*Lysinibacillus boronitolerans* YS11) were retrieved from the NCBI database. Quality-filtered reads were aligned to the reference‐genome sequence using Bowtie2. The abundance of relative transcript was shown by reads per kilobase of the exon sequence per million mapped sequence reads (RPKM) defined as total exon reads/(mapped reads in millions × exon length in kilobases). Metabolic pathways were analyzed based on KEGG pathway analysis and BLAST alignment with proteins using CLRNASeq program provided by Chunlab.

### Isolation procedure for strain AK13

To attain bacteria native to the environment of *L. boronitolerans* YS11, soil sample was amassed from the rhizosphere of *Miscanthus sacchariflorus* near Seongbukcheon in South Korea (37° 34′ 44.9″ N 127° 01′ 28.8″ E) (Lee et al. 2017). A 1 g of soil sample was prepared and washed with PBS (pH 7.5). It was then spread onto pH 13 adjusted LB agar. Plates were cultured overnight at 30 °C. Colonies that appeared on the agar plate were isolated and single colonies were roughly cocultured with YS11 in LB medium with three replicates to observe any difference in optical density (OD) compared to YS11 single culture. Cells were grown in PVC 96-well microtiter plates (BD Biosciences) at 30 °C and measured optical density at 595 nm (OD_595_) using biophotometer (Eppendorf, Germany). An isolate that showed a bump that had more than 1% increase in OD_565_ value in the growth curve during coculture was observed and named as AK13.

### Phylogenetic analysis of 16S rRNA gene sequence

The 16S rRNA gene of newly isolated alkaliphilic strain AK13 was amplified using universal bacterial 16S rRNA gene primers 27F (5′-AGAGTTTGATCMTGGCTCAG-3′) and 1492R (5′-TACGGYTACCTTGTTACGACTT-3′). Polymerase chain reaction (PCR) was conducted with the following cycling conditions: 94 °C for 90 s followed up by 25 cycles of 94 °C for 45 s, 58 °C for 45 s, and 72 °C for 45 s, and finally an extension step at 72 °C for 5 min. Sequence similarity with other bacteria was determined with EzTaxon program. A neighbor-joining tree was constructed using a distance matrix calculation method in MEGA 7. Bootstrapping was conducted with 1000 iterations. The fasta file of 16S rRNA gene sequence of strain AK13 was deposited at GenBank database (MK517564).

### Cocultivation for *L. boronitolerans* YS11 and *Bacillus* sp. AK13

Strain YS11 was incubated overnight in pH 6.8 LB broth while strain AK13 was cultured in pH 8 LB broth at 30 °C overnight. Cells were then washed twice with PBS. For coculture of YS11 plus AK13, 1 × 10^6^ CFU/ml of overnight isolates were inoculated into YL medium composed of .4% yeast extract and .34% calcium-acetate at 1:1 ratio. For single culture of YS11 and AK13 each, 2x10^6^ CFU/ml of overnight isolates were inoculated into YL medium. They were incubated at 30 °C with shaking at 220 rpm.

The growth rate of coculture and single culture was measured by counting colony-forming unit (CFU). Normal neutral LB agar was used as a selective medium for strain YS11 while LB agar with pH 12 was used as a selective medium for alkaliphilic strain AK13. Alteration of pH and unbound calcium ion concentration were measured using a pH electrode (Thermo Fisher Scientific, USA) and a calcium-ion selective electrode (ISE) (Thermo Fisher Scientific, USA), respectively. The 7 ml volume of sub-samples were removed from the master volume, over time for 24 h. For these sub samples, organic matters were removed by centrifugation at 4000 rpm for 10 min prior to measurements. These experiments were conducted in triplicates.

For coculture experiment in agar plates, two types of modified B4 medium, CaAc medium and NaAc medium, were used. Then 20 µl of each YS11 and AK13 overnight cultures (1 × 10^6^ CFU/ml) were spotted on the edge and opposite sides of the plate and then cultured at 30 °C for 24 h.

### FE-SEM and FTIR for morphological analysis of CaCO_3_

Mineral from YS11 single culture or YS11 plus AK13 coculture in YL medium was dried in an oven drier at 100 °C overnight prior to FE-SEM analysis. The sample was placed onto a carbon tape for 1 h to be dried. After blowing unattached particles with nitrogen gas, samples were Pt-coated and analyzed with a Quanta 250 FEG FE-SEM (FEI, USA). For FTIR analysis, an oven dried sample was resuspended in distilled water and analyzed using ATR method in FTIR (Agilent).

### pH measurement in pH increasing condition

To determine pH increasing mechanisms in various conditions, 10^6^ CFU/ml of overnight YS11 was inoculated into media consisting of either .8% yeast extract or .8% nutrient broth or MSB basal medium each supplemented with .1% pyruvate, .1% l-serine, or .1% pyruvate plus .1% l-serine. Total of 50 ml volume culture was incubated in 100 ml volume of Erlenmeyer flasks. OD_600_ and pH were measured in 24-h interval during their incubation at 30 °C with agitation at 220 rpm. 7 ml volume of sub samples were taken out from three replicates and measured right away. For the optical density measurement, 10^−1^ dilutions were done for each sample.

### Ammonia measurement assay

Ammonia concentration during incubation of YS11 in YL medium was measured using ammonia assay kit (Sigma-Aldrich). Briefly, supernatant (50 µl) of YS11 cultured in YL medium was mixed with ammonia assay reagent (500 µl) containing α-ketoglutaric acid and NADPH. Then, 5 µl of l-glutamate dehydrogenase was added to each sample and incubated for 5 min at 30 °C. The absorbance of each solution was measured at wavelength of 340 nm.

### CLSM analysis to examine biofilm and calcium carbonate development

Biofilms from YS11 and YS11 plus AK13 were stained for 30 min with FilmTracer™ SYPRO^®^ Ruby biofilm matrix dye at room temperature and visualized using CLSM (LSM700; Carl Zeiss, Jena, Germany). FilmTracer™ SYPRO^®^ Ruby biofilm matrix stained biofilm cells were used to obtain confocal images under red fluorescent light (excitation wavelength: 450 nm, emission wavelength: 610 nm). Calcium carbonate was visualized under blue fluorescent light by reflection signal of excitation between 483 and 493 nm (Bai et al. 2017). Both biofilms and precipitated calcium carbonate were evaluated for height and density of morphology (C-Apochromat 40×/1.20 W Korr M27; Carl Zeiss).

### Biofilm formation assay

Overnight cultures of YS11 and AK13, with the same condition mentioned in cocultivation section, were washed with room-temperature PBS twice at room-temperature. Overnight cells were then inoculated into 1 ml volume of YL medium to contain 2 × 10^6^ CFU/ml of strain YS11 or mixture of YS11 and AK13 at cell density of 1 × 10^6^ CFU/ml each in 48-well microtiter plates. The volume of the cells inoculated was measured by converting OD_600_ value of the overnight cells and converting them to the standard CFU/ml graph. Cell inoculated microtiter plates were then incubated at 30 °C with different time intervals (24 h, 48 h, 72 h) in static condition. Biofilms were stained with crystal violet dye using crystal violet staining assay (O’Toole 2011). Crystal violet attached to biofilms was dissolved in 95% ethanol solution. The optical density at 595 nm (OD_595_) was then measured for biofilm quantification using a multi-detection microplate photometer (HIDEX Sense, Finland).

### FAME analysis

Fatty acids from YS11 cultured in NaAc and CaAc media were extracted. Fatty acids were then saponified and methylated to be transformed into fatty acid methyl esters (FAMEs). At first, the bacterial cells washed twice with PBS, and were harvested with centrifugation. The cells were resuspended in 1 ml of saponification reagent into 15 ml falcon tube. This was vortexed for 30 s and heated at 100 °C for 5 min. The cells were once again vortexed for 10 s and heated at 100 °C for 25 min. Then, they were heated at room-temperature for 1 min and 2 ml of methylation reagent were added. This was vortexed for 10 s and heated at 80 °C for 10 min. The solution was cooled at room-temperature for 1 min. 1.25 ml of extraction solvent was added and vortexed for 10 min where the solution divides into two layers. The upper later was transferred into new 15 ml falcon tube. 3 ml of base wash solution was added and vortexed for 5 min. The upper layer of the solution was finally transferred into GC vials. These extracted FAMEs were analyzed using an Agilent 7890 GC system with a flow ionization detector and an HP-Ultra-2 capillary column (crosslinked 2.5% phenylmethyl silicone, 25 m, 200 mm i.d., film thickness: .33 mm). MIDI-2000 calibration standard was used to calibrate FAME values. FAMEs were identified and qualified using the Sherlock 6.0B MIDI software according to their equivalent chain value.

### FE-SEM/EDX analysis for CaCO_3_ nanoparticle formation

YS11 was incubated in CaAc medium for 6 and 12 h at 30 °C prior to FE-SEM and EDS analyses. Cells were fixed first with low-strength Karnovsky’s solution (2% paraformaldehyde, 2.5% glutaraldehyde, and .1 M phosphate buffer, final pH 7.2) for 2 h. Secondary fixation was done using 2% osmium tetroxide solution for 2 h. These fixed samples were gradually dehydrated with ethanol (30%, 50%, 70%, 100%) for 10 min each and placed onto aluminum stub for 4 days to be dried at room-temperature. These samples were then coated with platinum and analyzed using field-emission scanning electron microscope Quanta 250 FEG (FEI, USA) and energy dispersive X-ray spectrometer (EDS).

### Bacterial strains culture deposition number

A strain YS11 culture was stored in Agricultural Culture Collection (KACC) under number KACC 81048BP. A strain AK13 culture was stored in KACC with a deposition number of KACC 81070BP.

### Nucleotide and SRA sequence accession number

The complete genome sequence of *Lysinibacillus boronitolerans* YS11 has been deposited in NCBI under the GenBank accession number CP026007.1. The RNA-seq of *Lysinibacillus boronitolerans* YS11 has been deposited in NCBI under the SRA number SRR8083459.

## Results

### Complete genome sequencing of CaCO_3_ precipitating bacterium YS11

**Fig. 1:**
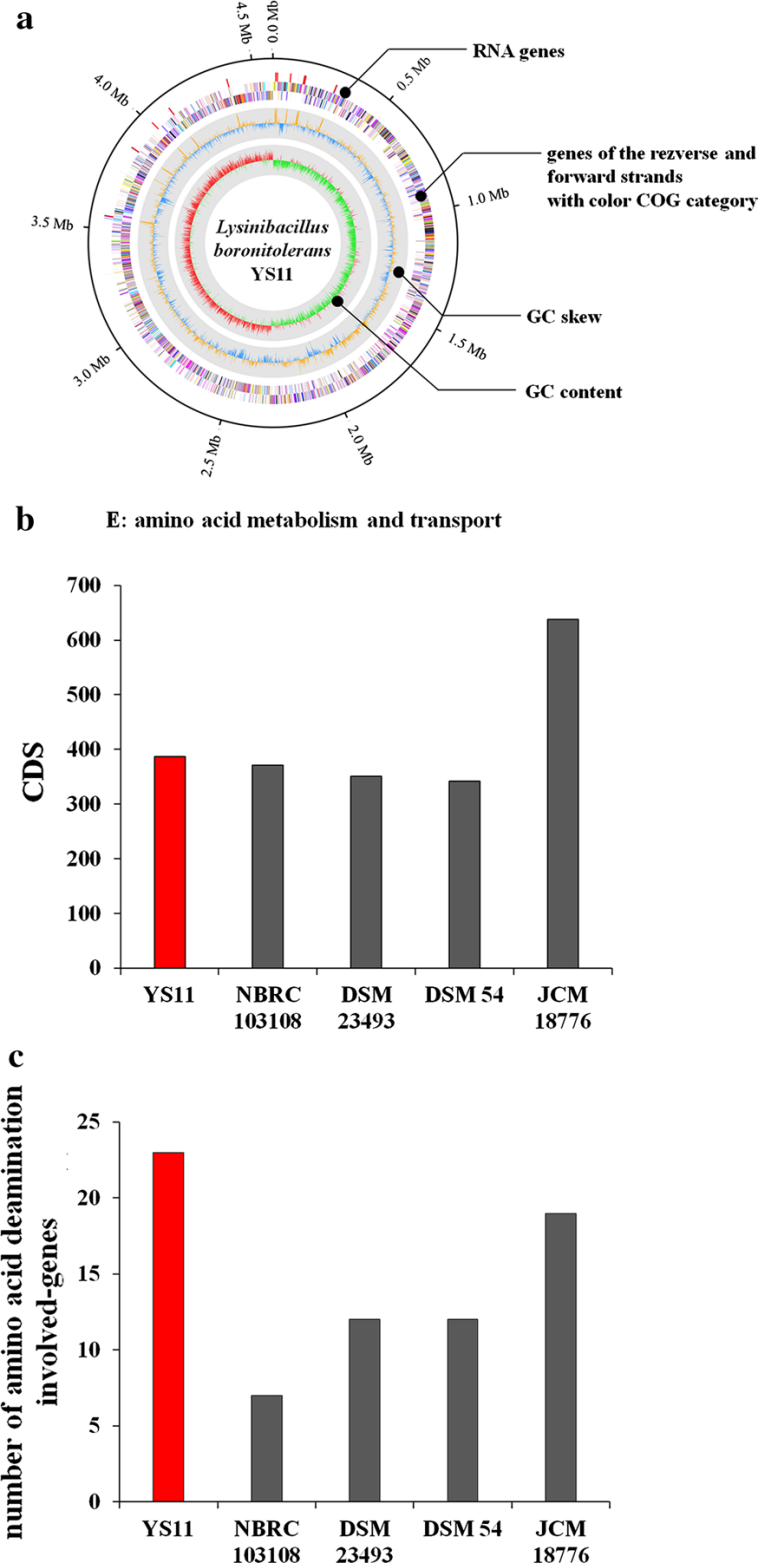
**a** Circular genomic map of *Lysinibacillus boronitolerans* YS11. From the outside to the center are RNA genes, genes of the reverse strand, genes on the forward strand, GC ratio, and GC skew. Genes are colored according to their COG category. **b** Number of CDSs belonging to amino acid metabolism and transport group from YS11 with other *Lysinibacillus* species including *L. boronitolerans* NBRC 103108^T^, *L. macroides* DSM 54 ^T^, *L. xylanilyticus* DSM 23493 ^T^, and *L. pakistanensis* JCM 18776 ^T^. **c** COG category of *Lysinibacillus* species. **d** Quantification of CDSs involved in deamination of amino acids among amino acid metabolism and transport groups from *Lysinibacillus* species

Whole genome sequencing of *Lysinibacillus* sp. YS11 previously reported for its non-ureolytic calcium carbonate precipitation (Lee et al. 2017) was conducted in this study. A complete genome comprising 1 circular chromosome with the genome size of 4,584,915 base pairs and GC content of 37.70% was obtained (Fig. 1a). Until now, a total of 111 genomes from species belonging to *Lysinibacillus* genus have been sequenced, including a CCP strain *Lysinibacillus sphaericus* LMG 22257 (Yan et al. 2017). The genome of *Lysinibacillus* sp. YS11 and genomes of other *Lysinibacillus* species, including *L. boronitolerans* NBRC 103108, *L. macroides* DSM 54, *L. xylanilyticus* DSM 23493, and *L. pakistanensis* JCM 18776, was compared (Table 1). Average Nucleotide Identity (ANI) of strain YS11 with other species belonging to *Lysinibacillus* genus revealed that YS11 belonged to *boronitolerans* species, showing ANI of 99.87% (Table 2). The genome size of YS11 was 4.584 kb, was 99.9% the size of *L. boronitolerans* NBRC 103108 (4.564 kb). *L. boronitolerans* YS11 possessed the highest number of tRNA genes (34) and rRNA genes (107) among the other four species. Similar to results from ANI analysis, analysis for the presence and absence of genes also verified that YS11 was closest to *L. boronitolerans* NBRC 103108 and the farthest from strain JCM 18776 (Additional file 1: Fig. S1A). YS11 also retained similar distributions of genes in COG category with strain DSM54, in which the most abundant genes were affiliated to amino acid metabolism and transport (E) (Additional file 1: Fig. S1B). Except *L. pakistanensis* JCM 18776, strain YS11 retained most abundant number of CDS related to amino acid metabolism and transport (E) COG group (Fig. 1b). Genes involved in ammonia production for possible alkaline generating pathway were searched in the genome of YS11. There are total of 35 genes that were directly involved in ammonia production (Table 3). When the number of genes involved in deamination of amino acids in the genome of *L. boronitolerans* YS11 was compared to that of NBRC 103108, a phylogenetically similar species, YS11 possessed about 3 times more genes of amino acid deaminases, suggesting species specific characteristics of basic compound production (Fig. 1c). Along with strain NBRC 103108, other *Lysinibacillus* species also showed smaller numbers of amino acid deaminases (Fig. 1c).

**Table 1:** Genomic features of *L. boronitolerans* YS11 compared with other *Lysinibacillus* species including *L. boronitolerans* NBRC 103108^T^, *L. macroides* DSM 54^T^, *L. xylanilyticus* DSM 23493^T^, and *L. pakistanensis* JCM 18776^T^

Features	Values				
	YS11	NBRC 103108	JCM 18776	DSM 54	DSM 23493
Genome size (Mb)	4.58	4.56	5.01	4.87	5.22
GC content (%)	37.7	37.6	36.3	37.7	36.5
Contigs	1	81	73	15	13
CDS	4602	4576	8211	4713	4884
rRNAs	34	6	5	9	20
tRNAs	107	52	74	86	100

**Table 2:** Average nucleotide identity (ANI) of strain YS11 with other *Lysinibacillus* species

Strain YS11	Average nucleotide identity (%)
*Lysinibacillus boronitolerans* NBRC 103108^T^	99.87
*Lysinibacillus pakistanensis* JCM 18776^T^	98.93
*Lysinibacillus macroides* DSM 54^T^	98.16
*Lysinibacillus xylanilyticus* DSM 23493^T^	97.27

**Table 3:** Genes involved in production of ammonia and deamination of amino acids

Coordinates	Strand	Product	Gene name
266,921–268,435	+	Histidine ammonia-lyase	*hutH*
318,892–320,310	−	Putative amidase AmiD	
435,365–436,537	+	Lysine 6-dehydrogenase	
535,842–537104	−	Phenylserine dehydratase	
1,039,715–1,040,377	+	Probable l-serine dehydratase, beta chain	*sdaAB*
1,040,392–1,041,300	+	Probable l-serine dehydratase, alpha chain	*sdaAA*
1,101,650–1,102,153	+	Probable chemoreceptor glutamine deamidase CheD 1	
1,318,421–1,319,788	−	Guanine deaminase	*guaD*
1,430,688–1,431,656	−	l-Asparaginase	
1,547,319–1,548,554	+	Allantoate deiminase	
1,581,552–1,582,922	+	Ethanolamine ammonia-lyase heavy chain	
1,582,932–1,583,942	+	Ethanolamine ammonia-lyase light chain	
1,614,306–1,615,679	−	NADP-specific glutamate dehydrogenase	
1,616,318–1,617,646	+	Probable d-serine dehydratase	
1,804,351–1,805,055	+	Glucosamine-6-phosphate deaminase	
1852010–1852543	+	Peroxyureidoacrylate/ureidoacrylate amidohydrolase RutB	
2,097,252–2,098,670	+	Aspartate ammonia-lyase	*aspA*
2,100,543–2,100,845	+	Urease subunit gamma	*ureA*
2,100,861–2,101,229	+	Urease subunit beta	
2,101,226–2,102,941	+	Urease subunit alpha	*ureC*
2,423,272–2,424,252	−	Meso-diaminopimelate d-dehydrogenase	
2,687,063–2,688,808	−	Adenine deaminase	*ade*
2,743,214–2,744,329	+	Cystathionine beta-lyase MetC	
2,850,380–2,851,474	−	Leucine dehydrogenase	
2,897,281–2,898,246	−	Glutaminase 1	*glsA*
2,913,990–2,915,093	−	Aminomethyltransferase	*gcvT*
3,177,391–3,178,323	−	Porphobilinogen deaminase	
3,406,895–3,408,010	−	Alanine dehydrogenase	*ald*
3,539,367–3,540,581	−	Diaminopropionate ammonia-lyase	
3,564,845–3,566,089	−	Catabolic NAD-specific glutamate dehydrogenase RocG	
3,575,261–3,576,433	+	Cystathionine beta-lyase PatB	
3,688,923–3,690,383	−	Putative amidase AmiC	
3,856,477–3,857,190	−	Glucosamine-6-phosphate deaminase	*nagB*
3,991,464–3,993,185	−	Putative adenine deaminase PTO1085	
4,002,226–4,002,909	−	Phosphoribosylformylglycinamidine synthase subunit PurQ	

### Alkaline generation by YS11 is suitable for CaCO_3_ precipitation

**Fig. 2:**
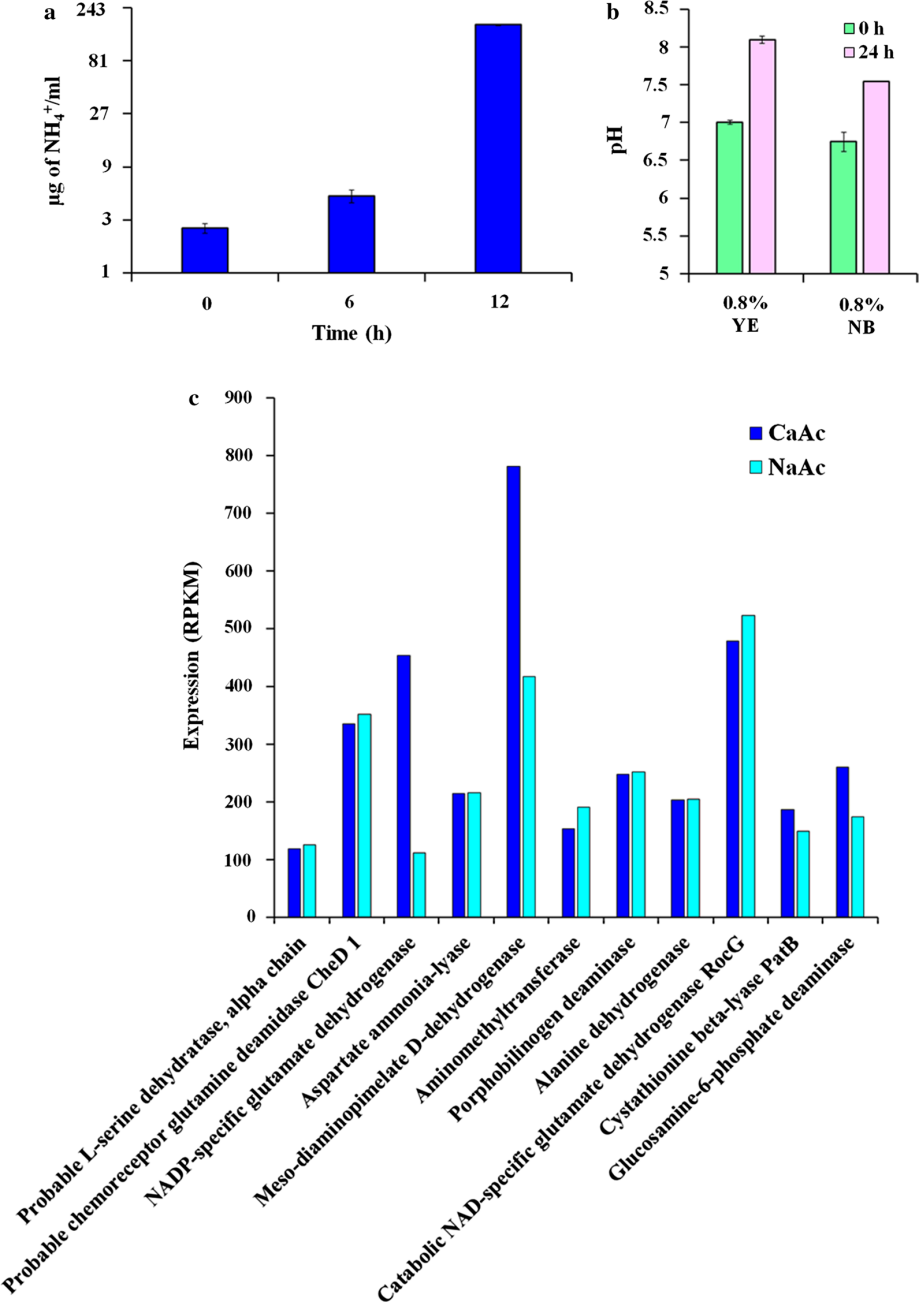
**a** Quantification of ammonia production from supernatant of YS11 cultured in YL medium. **b** pH increases by YS11 in amino acid rich media (.8% yeast extract or .8% nutrient broth). **c** Expression of ammonia releasing genes from YS11 that exhibited RPKM value higher than 100

Ammonia concentration was measured during the growth of YS11 in YL medium. The increase of ammonia concentration in accordance with pH increase suggested that ammonia production might be a major way for alkaline generation (Fig. 2a). Thus, alkaline generating pathways of non-ureolytic YS11 were analyzed using phenotypic and genomic approaches. Since yeast extract in YL medium is abounded with amino acids, pH change in two kinds of rich medium culture each supplemented with .8% yeast extract and .8% nutrient broth was measured (Fig. 2b). YS11 could increase the pH in both conditions, highly suggesting that deamination of rich amino acids provided by yeast extract in the medium was the key factor in ammonia production during this condition. Since yeast extract is an undefined complex component known to attain rich amino acids, transcriptomes during pH increasing conditions of CaAc and NaAc were analyzed. When data were trimmed with RPKM expressions higher than 100, genes involved in deamination of the following eight compounds were expressed: l-serine, glutamine, glutamate, aspartate, meso-diaminopimelate, porphobilinogen, alanine, cystathionine, and glucosamine-6-phosphate (Fig. 2c).

### Increase in pH level by YS11 induces interspecies interaction with alkaliphilic bacterium

In order to observe interspecies interaction in biofilm mediated calcium carbonate formation, additional isolation from where YS11 had been found was conducted to explore species that is in cooccurrence with YS11. Alkaliphilic strains were targeted for screening procedure as *L. boronitolerans* YS11 promotes alkali condition during the growth, and strain AK13 was isolated. In the procedure, bacterial isolates with alkaliphilic properties were screened in pH 13 adjusted LB medium culture. After incubation, colonies formed in LB medium were further tested for coculture with strain YS11. The phylogenetic tree of strain AK13 was evaluated with 16S rRNA gene sequences, including strains showing high similarity (Additional file 1: Fig. S2A). Strain AK13 was phylogenetically affiliated with genus *Bacillus*. Its 16S rRNA gene sequence showed the highest similarity (99.88%) with that of *Bacillus hunanensis* JSM 091003^T^, *Bacillus oshimensis* DSM 18940^T^, and *Bacillus lehensis* MLB-2^T^. These 16S rRNA gene sequences of strain AK13 also shared high similarities with *Bacillus xiaoxiensis* JSM 081004^T^ (99.25%) and *Bacillus patagoniensis* DSM 16117^T^ (99.13%). Strain AK13 was able to grow well without any inhibition at alkaline pH range from pH 8 to pH 11 compared to its growth in pH 7, showing an optimal growth at pH 8 (OD_600_ ~ .65). Its growth was inhibited at pH 7 and pH 12. AK13 strain was unable to grow at pH 6 (Additional file 1: Fig. S2B). This implies that AK13 is an alkaliphilic bacterium (with optimal growth in alkaline condition, unable to grow or its growth is hampered in neutral condition). On the other hand, YS11 showed optimal growth rate at neutral pH 7. YS11 strain was able to grow at pH 6 and at pH 9, but not at pH 10 and pH 11 (Additional file 1: Fig. S2B).

**Fig. 3:**
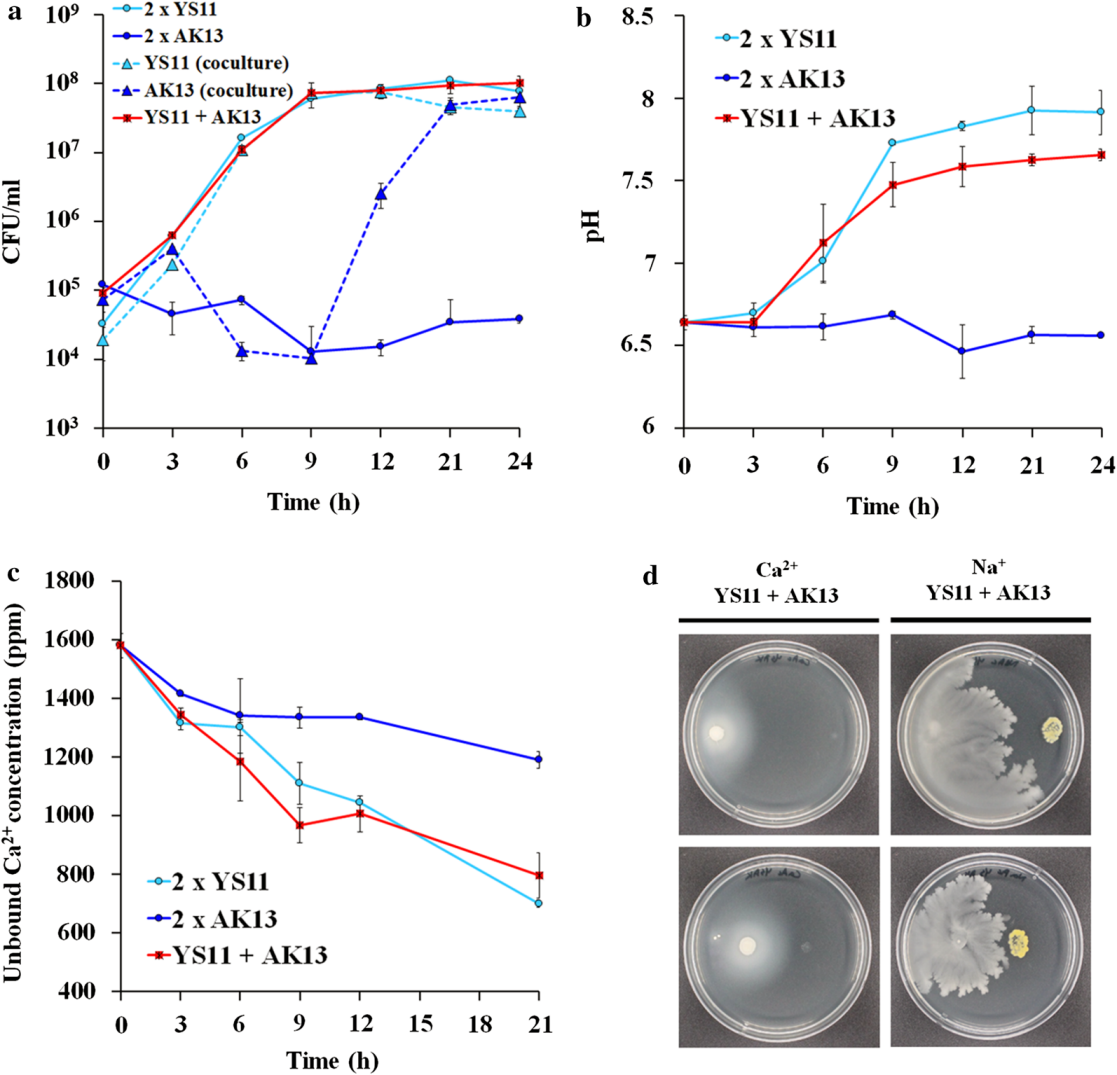
Growth, pH changes, and calcium utilization in MICP-inducing conditions with YS11, AK13 alone, and YS11 + AK13 coculture. **a** Based on CFU growth of YS11, AK13, and YS11 + AK13, the growth of AK13 is facilitated by coculture with YS11. **b** pH changes in YS11, AK13 single culture, and YS11 + AK13 coculture are shown. **c** Unbound Ca^2+^ concentrations in supernatants of YS11, AK13 single culture, and YS11 + AK13 coculture are measured. **d** Induced growth of AK13 from coculture with YS11 in agar plate cultures

Further experiments were performed to observe interaction among these two different bacteria during cocultivation. These two strains were cocultured in neutral growth medium of pH 6.8 to observe interspecies interaction in both liquid culture and agar plate culture (Fig. 3a, d). Yellow colony of AK13 also appeared in the agar plate culture when YS11 and AK13 were each spotted at the edge of the opposite side of the plate. To identify the co-cultivation more specifically, each species’ growth in pH varying LB selective medium, pH change, and unbound calcium concentration were measured. The growth of alkaliphilic AK13 in single culture was not detected throughout the experiment. Strain YS11 single culture grew to cellular density up to 10^8^ CFU/ml, reaching its stationary phase at 9 h after incubation (Fig. 3a). On the other hand, when these two bacteria were cultured together at 1:1 ratio of initial cellular density of 10^6^ cell/ml, strain AK13 could retrieve its growth starting from 9 h after incubation (Fig. 3a). This result suggests pH increase by strain YS11 enables the growth of alkaliphilic AK13 during their cooccurrence. The pH of the coculture and that of YS11 single culture were both increased along with bacterial growth. The pH increases in coculture (up to pH 7.6) was less than that of YS11 alone (up to pH 7.9) (Fig. 3b). Unbound calcium ion concentration gradually decreased with YS11 alone and YS11 plus AK13 coculture, although the coculture showed faster decrease in calcium concentration (Fig. 3c). No prominent decrease in calcium ion concentration was detected for AK13 single culture since there was no growth. However, a slight decrease of approximately 300 ppm of calcium ions was found. This might be due to charge–charge interaction of initially inoculated cell wall and calcium ion (Fig. 3c). For the alkali generating mechanism by YS11, deamination of amino acids is highly speculated as strain YS11 pertains to deamination pathways, contributing to its alkali generating characteristic.

### Co-occurrence alters the appearance of calcium carbonate precipitation

**Fig. 4:**
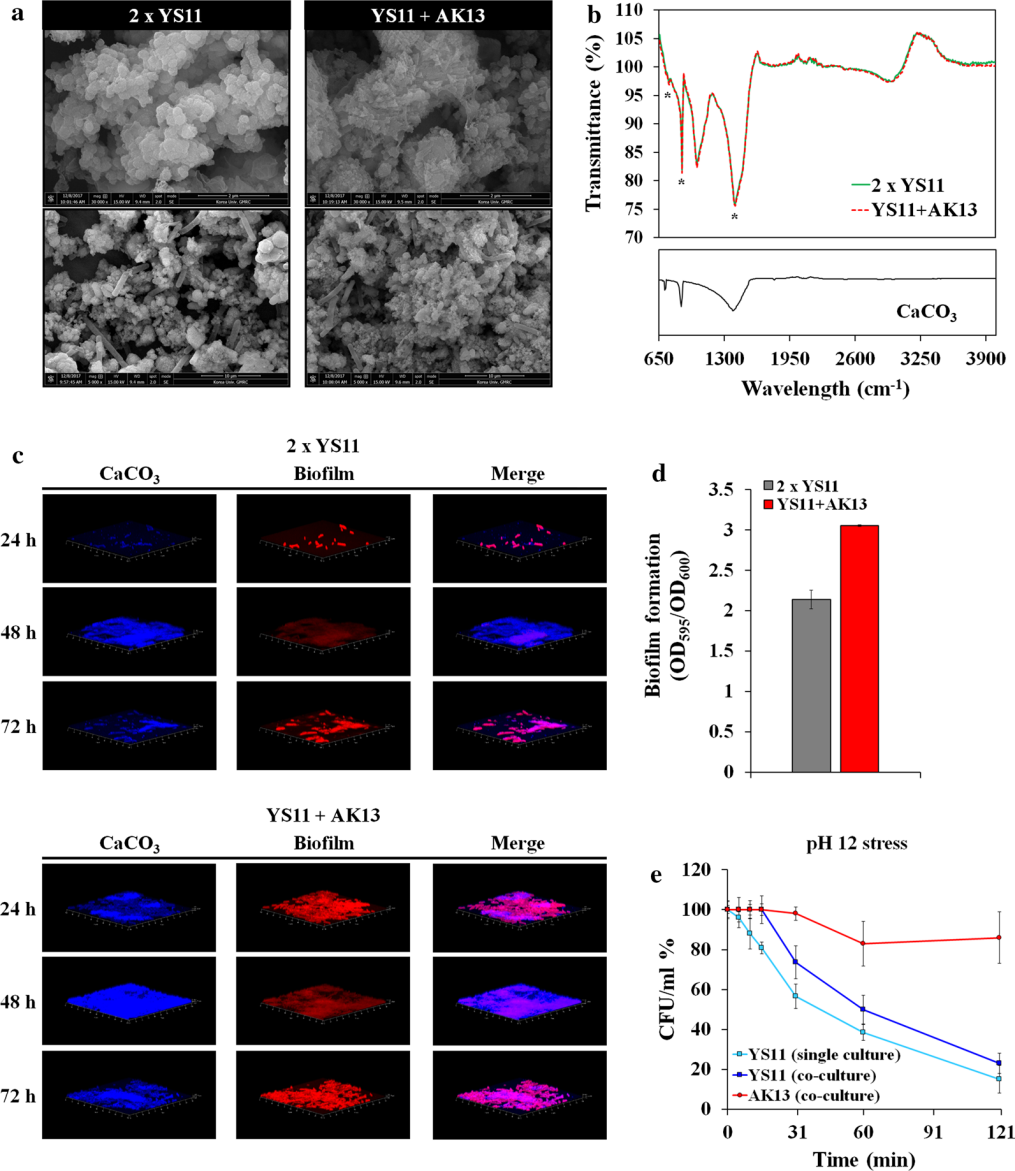
**a** Field emission scanning electron microscopy (FE-SEM) of calcium carbonate precipitated by YS11 single culture and YS11 + AK13 coculture (×30,000 and ×5000 magnification). **b** FTIR analysis of precipitated calcium carbonate. **c** CLSM image of biofilm and calcium carbonate produced by YS11 single culture. **d** Crystal violet quantification of biofilm formation in single culture or YS11 + AK13 coculture. **e** Killing curve assay of YS11 alone, AK13 alone, and YS11 plus AK13 under pH 12

After monitoring the growth, pH change, and calcium ion concentration, cultured cells were visualized for precipitated calcium carbonate minerals. It has been characterized that *L. boronitolerans* YS11 precipitates calcium carbonate when calcium source is provided in the medium (Lee et al. 2017). Resultant calcium carbonate was confirmed from both YS11 culture alone and YS11 plus AK13 coculture condition (Fig. 4a, b). MICP induced by YS11 in YL medium resulted in round shaped mineral aggregates whereas MICP induced by mixed culture depicted smaller particle size and more compact form (Fig. 4a). Besides such difference in mineral shape, the coculture showed distinct formation of EPSs within calcium carbonate-cell clusters (Fig. 4a). Minerals precipitated in both cultures were verified to be calcium carbonates as the FTIR analysis showed three peaks of samples identical to reference calcium carbonate peaks (Fig. 4b).

To verify relations between EPSs and calcium carbonate generation, cells were incubated in static condition for biofilm and calcium carbonate development periodically. Image analysis by CLSM showed similar results (Fig. 4c). Both YS11 single culture and YS11 plus AK13 coculture were in developmental stage of biofilm in 24 h, matured biofilm in 48 h, and dispersed form in 72 h. In all stages, biofilm formed under YS11 plus AK13 coculture condition was much higher (Fig. 4c). Along with biofilm formation, much more biofilm bound calcium carbonate was observed in the coculture (Fig. 4c). Quantification of biofilm formation was performed after crystal violet staining to examine equivalent results as CLSM images. Accordingly, biofilm formation in coculture was measured to be 1.5-fold higher than that in YS11 single culture (Fig. 4d). Enhanced biofilm formation in coculture by strain AK13 might give nucleation site for calcium carbonate, suggesting more surface-calcium carbonate bound cells.

Survival of YS11 in sole culture and coculture were compared under alkaline pH of 12. The survival of YS11 from the coculture was improved (Fig. 4e). This suggests that altered EPS composition and quantity of biofilm formation might have contributed to the improvement in the survival of YS11 when it is cocultured with AK13. CCP bacteria are often applied inside building materials such as concrete for repairment of crack initiations (Lee and Park 2018). Because the pH of the concrete matrix is up to 13, our result suggests that increased survival from the co-occurrence of YS11 and AK13 could also show enhanced healing rate of cracks when applied into cementitious materials.

### Modification of membrane rigidity during calcium carbonate precipitation

**Fig. 5:**
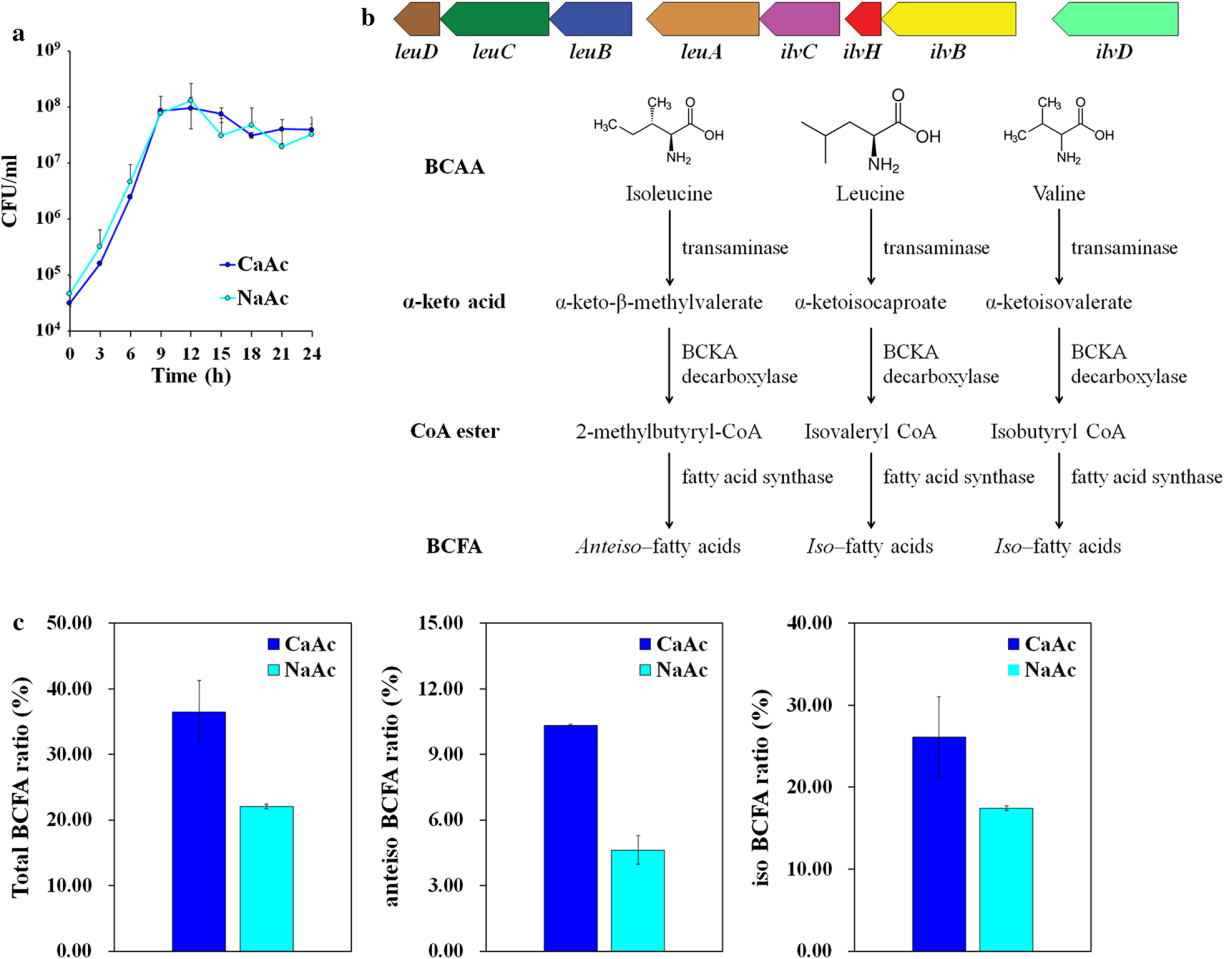
**a** Growth curve of YS11 in calcium-rich condition (CaAc) and calcium-poor condition (NaAc). **b** Schematic view of upregulated branched chain amino acid (BCAA) and branched chain fatty acid (BCFA) synthesis in CaAc. **c** Modified BCFA ratio in CaAc compared to that in NaAc

**Fig. 6:**
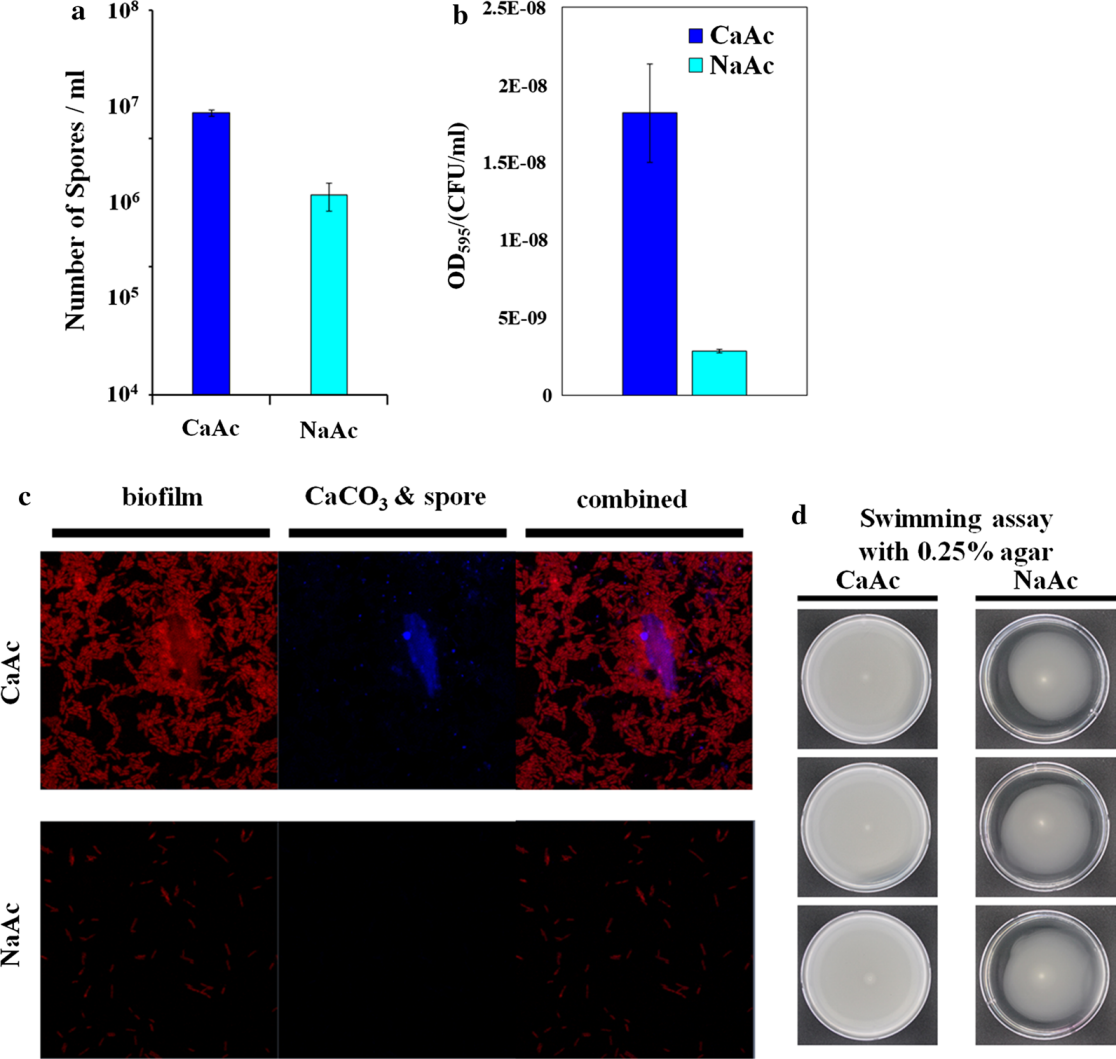
**a** Enhanced spore formation of YS11 in calcium-rich condition. **b** Increased biofilm formation in calcium-rich condition. **c** CSLM image of increased biofilm (red) formation in calcium-rich condition (blue color indicates calcium carbonate mineral). **d** Increase in swimming motility in calcium-rich condition compared to that in calcium-poor condition

The growth curve of YS11 in CaAc medium and NaAc medium showed similar growth rates (Fig. 5a). To determine changes in physiology of YS11 during calcium carbonate precipitation when Ca^2+^ was near, RNA-seq was conducted for mid-exponentially grown cells from both NaAc and CaAc conditions (Table 4). Interestingly, upregulation of branched chain amino acid (BCAA) and branched chain fatty acid (BCFA) synthesis was noticeable in transcriptomics of calcium carbonate precipitation (CaAc medium) compared to that of the control (NaAc medium). YS11 retained all genes involved in both BCAA and BCFA synthesis in one operon (Fig. 5b). BCAA synthesis pathway is directly connected to BCFA pathway where BCFA are produced as cellular components (Fig. 5b). Furthermore, genes involving multi-drug efflux pump, membrane protein related genes, and membrane protease-involved genes were also upregulated when Ca^2+^ was provided (at CaAc condition) (Table 4). These results suggest modification of membrane during MICP. Thus, fatty acid methyl esters (FAMEs) of YS11 from NaAc and CaAc cultures were quantified to determine any modification in BCFA composition. Noticeably higher value in total BCFA ratio including anteiso BCFA and iso BCFA ratios were measured under CaAc condition compared to that under NaAc condition after 12 h of culture (Fig. 5c). This result demonstrates alteration in phenotype of membrane rigidity from the presence of Ca^2+^. However, the BCFA ratio in 6 h CaAc condition was unsubstantial compared to that in 6 h NaAc condition. Although gene expression at CaAc condition showed considerable difference compared to that at NaAc condition at 6 h, protein difference and phenotypical changes were only evident from 12 h. For the first time, we show genomic and phenotypic characteristics of CCP bacteria during CaCO_3_ precipitation through differential gene expression and FAME analyses. It was questionable whether calcium carbonate crystals were present during 6 h incubation where major transcriptomic changes were observed. FE-SEM and EDS obtained after 6 h of incubation in CaAc medium confirmed the presence of calcium carbonate (Additional file 1: Fig. S3). Presence of Ca^2+^ also affects phenotypes of *Lysinibacillus boronitolerans* YS11. Spore formation and biofilm formation were all increased in CaAc compared to those in NaAc (Figs. 6a, b). The increase of biofilm formation in CaAc was also observed in CLSM image where the glass surface was covered up by biofilms under CaAc condition compared to scattered biofilm under NaAc condition (Fig. 6c). Swimming motility was also measured under CaAc and NaAc conditions. Swimming of YS11 in Ca^2+^-rich condition was much higher than that under NaAc condition. It covered all area of the petri dish (Fig. 6d). Ca^2+^-specific differences in various phenotypes demonstrate that strain YS11 shows responses corresponding to Ca^2+^. Further experiments are needed to determine the mechanisms underlying these differences.

**Table 4:** Upregulated and downregulated genes in CaAc and NaAc condition (gene expression ≥ 100, fold change ≥ 2, and *p* value ≤ .05)

Locus taq	Gene symbol	Product	Strand	CaAc RPKM	NaAc RPKM	CaAc/NaAc
Genes upregulated by high Ca^2+^						
Branched chain amino acid synthesis						
LBYS11_13585		Acetolactate synthase small subunit	−	144.04	14	10.29
LBYS11_13570	*leuB*	3-Isopropylmalate dehydrogenase	−	124.41	12.67	9.82
LBYS11_13580		Ketol-acid reductoisomerase	−	263.2	27.42	9.6
LBYS11_13560	*leuD*	3-Isopropylmalate dehydratase small subunit	−	116.42	12.75	9.13
LBYS11_13565	*leuC*	3-Isopropylmalate dehydratase large subunit	−	144.46	17.55	8.23
LBYS11_13575		2-Isopropylmalate synthase	−	165.84	20.78	7.98
LBYS11_13590	*ilvB*	Acetolactate synthase large subunit IlvB1	−	131.49	18.13	7.25
LBYS11_13595	*ilvD*	Dihydroxy-acid dehydratase	−	223.6	41.14	5.44
Branched chain fatty acid synthesis						
LBYS11_00205		Hypothetical protein (NCBI blast: 3-oxoacyl-(Acyl-carrier-protein (ACP)) synthase III)	+	1150.45	113.55	10.13
LBYS11_00220		Phosphopantetheine-binding protein	+	1044.75	125.49	8.33
LBYS11_00200		Hypothetical protein: holo-[acyl-carrier-protein] synthase	+	956.13	115.3	8.29
LBYS11_00230		Hypothetical protein: holo-[acyl-carrier-protein] synthase	+	1028.85	125.53	8.2
Lysine synthesis						
LBYS11_00215	*lysA*	Diaminopimelate decarboxylase	+	1089.26	118.57	9.19
Might serve as radioprotective agent						
LBYS11_06125		Glutathionylspermidine synthase	+	315.38	82.76	3.81
Protease involved genes						
LBYS11_00265		Uncharacterized zinc protease YmfH	+	754.93	96.72	7.81
LBYS11_00260		Zinc protease	+	685.49	90.94	7.54
LBYS11_06700		n|Uncharacterized protein YuaG	+	560.04	124.56	4.5
LBYS11_16935	*hflK*	FtsH protease activity modulator HflK|Protein HflK	−	605.83	146.1	4.15
LBYS11_16930	*hflC*	Protease modulator HflC|Protein HflC	−	887.2	231.43	3.83
LBYS11_17070	*sppA*	Signal peptide peptidase SppA|Putative signal peptide peptidase SppA	−	139.57	36.6	3.81
Multidrug efflux pump and swarming motility involved genes						
LBYS11_00190		Multidrug export protein AcrF Cobalt-zinc-cadmium resistance protein CzcA	+	336.58	42.35	7.95
LBYS11_00195		Swarming motility protein SwrC	+	800.18	106.83	7.49
LBYS11_00255		Hypothetical protein Multi antimicrobial extrusion protein (Na(+)/drug antiporter)	+	376.26	51.85	7.26
LBYS11_00250		Hypothetical protein Multi antimicrobial extrusion protein (Na(+)/drug antiporter)	+	265.05	37.79	7.01
TCA cycle involved genes						
LBYS11_00240		Acyl-CoA dehydrogenase	+	1676.26	217.77	7.7
LBYS11_07645		NADP-specific glutamate dehydrogenase	−	453.98	112.24	4.04
Hydantoinase						
LBYS11_08510		*N*-methyl hydantoinase	+	162.46	21.19	7.67
LBYS11_08515		Hydantoinase subunit beta	+	225.3	32.87	6.85
Membrane proteins						
LBYS11_13515		Uncharacterized membrane protein YceF	+	664.23	68.7	9.67
LBYS11_06695		Uncharacterized membrane protein YuaF	+	432.69	89.78	4.82
LBYS11_17065		RDD family protein|Uncharacterized membrane protein YteJ	−	362.02	93.05	3.89
LBYS11_20665		Hypothetical protein|UPF0699 transmembrane protein YdbS	−	148.94	39.48	3.77
Hypothetical proteins						
LBYS11_00210		Hypothetical protein	+	1190.22	124.33	9.57
LBYS11_00225		Hypothetical protein	+	1021.86	116.54	8.77
LBYS11_00235		Hypothetical protein	+	1193.34	140.21	8.51
LBYS11_00245		Hypothetical protein	+	1002.8	129.52	7.74
LBYS11_09000		Hypothetical protein	+	232.67	38.56	6.03
LBYS11_09005		Hypothetical protein	+	186.62	33.96	5.5
LBYS11_09815		Hypothetical protein	+	153.15	32.91	4.65
LBYS11_21200		Hypothetical protein	−	143.8	32.67	4.4
LBYS11_19760		Hypothetical protein|Uncharacterized protein YvlA	+	133.72	33.88	3.95
LBYS11_06120		Hypothetical protein|Uncharacterized serine-rich protein C21513	+	413.46	112.58	3.67
Genes downregulated by high Ca^2+^						
LBYS11_19235		Copper amine oxidase	−	63.11	224.53	.28
LBYS11_08750		Copper amine oxidase	+	52.07	239.12	.22
LBYS11_20870		Zinc transporter ZupT	−	133.27	521.26	.26
LBYS11_01430		Divalent metal cation transporter	−	8.88	211.74	.04
LBYS11_20140		Guanine/hypoxanthine permease	−	9.31	157.38	.06

## Discussion

CCP bacteria prevail in the environment. However, their ecological interactions between species remain to be examined in detail. Complete genome analysis for strain YS11 was performed. Through average nucleotide identity (ANI) analysis, this species belonged to *L. boronitolerans.* Upregulation of deaminase activities in pH increasing conditions from RNA-seq and ammonia production support that deamination of amino acids was a factor involved in alkaline generation by YS11. In oral cavity, alkaline generating pathways have been proposed to be arginine deaminase system or arginine decarboxylated agmatine deaminase system together with urea hydrolyses (Liu et al. 2012). To explore interspecies interaction of this alkaline generating strain YS11, isolation of additional bacteria was proceeded from the isolation spot of strain YS11 to identify bacteria in cooccurrence with YS11. Interestingly, an alkaliphilic bacterium *Bacillus* sp. AK13 whose growth was inhibited in neutral condition was isolated from the identical spot where neutrophilic strain YS11 was isolated. YS11 was able to facilitate the growth of AK13 by alkalization of neutral environment when they were cocultured. It is interesting to note that these two species that co-occur at the same habitat show very different degrees of optimal growth dependent on pH. Coculture of the two species promoted increased MICP within biofilm matrix. Structure analysis and quantification of biofilm and calcium carbonate also suggested that growths of both bacteria were enhanced in coculture condition. Our result of one alkali generating species enabling the survival of another alkaliphilic species suggests that species possessing ecologically different niches contributing to diverse environmental parameters can co-occur in same habitat by induction of an adequate environment by the other species. A recent study has investigated interspecies interaction among *Lysinibacillus fusiformis* M5 and *Bacillus subtilis* 168 (Gallegos-Monterrosa et al. 2017). It was found that hypoxanthine released from *L. fusiformis* induced architectural changes in biofilm of *B. subtilis*, thus altering wrinkle formation. Furthermore, transcriptomics under Ca^2+^ poor and Ca^2+^ rich conditions were conducted as to understand genetic characteristics and differential gene expressions during CaCO_3_ precipitation. Under Ca^2+^ rich condition, genes involved in branched chain amino acid and branched chain fatty acid syntheses were highly upregulated, including multi-drug efflux pump and membrane protein related genes, suggesting modification in membrane during Ca^2+^ contact. Various phenotypes (growth, biofilm formation, sporulation, swimming motility, and stress response) were altered under Ca^2+^ rich condition. Our study demonstrates that pH increase of *L. boronitolerans* YS11 enables the growth of *Bacillus* sp. AK13 dwelling in the same environmental niche. Such dual species interaction in the environment shapes CCP characteristics and leads to higher biofilm formation.

In this study, the interaction between two environmental isolates, alkali generating CCP bacteria *Lysinibacillus boronitolerans* YS11 and alkalitolerant *Bacillus* sp. AK13, dwelling at same location was examined. While neutral environment was a barrier for the growth of *Bacillus* sp. AK13, alkalization provided by *L. boronitolerans* YS11 enabled adequate living condition for AK13. Alkaline generation by bacteria has been observed many times. Recently alkaline generating pathways in oral bacteria have been identified through meta-omics approach (Edlund et al. 2015). According to that study, major pH increasing pathways for oral bacterial microbiome include the following: (1) arginine deaminase system, (2) urea transport and urease activity, (3) glutamate transport and glutamate dehydrogenase activity, (4) threonine deaminase activity, and (5) serine deaminase activity. Complete genome sequence of strain YS11 was obtained in this study to identify alkaline generation pathway leading to MICP. Strain YS11 contained many genes involved in deamination of amino acids (Table 3). Ammonia measurement in YL medium showed an increase in ammonia production, suggesting that strain YS11 could utilize amino acids provided by amino acid rich yeast extract (Fig. 2a, b). Transcriptomic analysis from RNA-seq at CaAc and NaAc conditions support that genes involved in deamination were expressed during pH increase in this complex media (Fig. 2c). Ammonia production from other amino acids deamination pathways, including glutamate dehydrogenase, arginine deaminase, and threonine deaminase, is highly possible based on genomic analysis of YS11 (Table 3). Co-occurrence of strain YS11 and alkaliphilic AK13 was thus enabled through deamination activities of YS11. The morphology of calcium carbonate produced from mixed culture was altered into compact shape and biofilm attached calcium carbonate production was higher from SEM and CLSM analyses (Fig. 4a, c). Co-occurrence of these two species also enhanced biofilm formation compared to single YS11 culture. These results suggest that alteration of EPSs components from dual species interaction can lead to formation of morphologically different calcium carbonate in coculture compared to single culture. Interestingly, AK13 in the biofilm only comprised of .05% whereas its biomass in agitation planktonic culture was 60% (data not shown). The phenotypic change in biofilm formation was significant even in miniscule population and the production of biofilm attached calcium carbonates increased accordingly (Fig. 3). Coculture with AK13 in return enhanced pH susceptibility of YS11. Strain YS11 was able to survive better when it was co-occurred with strain AK13 (Fig. 4d). These results demonstrate that bacteria having different physiological characteristics such as alkali generating feature and alkaliphilic feature can dwell collaboratively in nature and affect mineral formation and cell-surface attachment in nature. Ecological characteristics of alkali generating CCP bacteria in a par with an alkaliphilic bacteria were observed in this study. Nucleation effect of bacterial EPS has been accepted as a notion for bacterial MICP (Dupraz et al. 2009; Tourney and Ngwenya 2009; Zhu and Dittrich 2016). One study has explored interspecies interaction between *Lysinibacillus* species and *Bacillus* species and found that soil bacteria *L. fusiformis* can induce architectural changes in biofilm colonies when it is cocultured with *Bacillus subtilis* (Bilecen and Yildiz 2009). EPSs (exopolysaccharides) are crucial in shaping the morphology of calcium carbonate and its quantity because they lender nucleation sites for mineral precipitation. Thus, the property of EPSs holds great potential for calcium carbonate precipitation (Dupraz et al. 2009). To test surface chemical properties and attachment ability, EPS components should be analyzed.

Researches in biofilm have evolved to show major interest in microbiology recently. Because of the innate characteristic of biofilm as a sanctuary in various environmental conditions, biofilm possesses social interactions for multiple microbial populations, ranging from a single, a pair, to multispecies communities (Knoll 2003). These interactions between microorganisms forming a biofilm play a substantial role in this research field. They shape the development of these communities, make the community complex by intraspecies signaling, interspecies communications, or chemical cues derived from the metabolism of certain community members. It was pH signaling in this paper. Here, we demonstrate that interaction of two environmental bacterial interspecies in biofilm further influences mineral precipitation patterns on Earth surface. Further study on mixed culture MICP of biofilms will lead to improved understanding of biogenic carbonate precipitation in nature.

## Additional file

**Additional file 1: Fig. S1.** (A) Presence absence gene analysis of YS11 with other *Lysinibacillus* species including *L. boronitolerans* NBRC 103108^T^, *L. macroides* DSM 54^T^, *L. xylanilyticus* DSM 23493^T^, and *L. pakistanensis* JCM 18776^T^. (B) COG category of *Lysinibacillus* species. **Fig. S2.** (A) Phylogenetic neighbor joining tree of strain AK13. (B) pH dependent growth of alkalifying strain YS11 and alkaliphilic strain AK13. **Fig. S3.** FE-SEM and EDX analyses for nanoparticle calcium carbonate formed during early growth (6 h).

## Abbreviations

*L. boronitolerans*: *Lysinibacillus boronitolerans*
MICP: microbially induced calcium carbonate
CCP: CaCO_3_ precipitating
EPS: exopolysaccharide
LB: Luria-Bertani
PBS: phosphate buffered saline
CaAc: calcium acetate
NaAc: sodium acetate
CFU: colony-forming unit
RPKM: reads per kilobase of the exon sequence per million mapped sequence reads
OD: optical density
FE-SEM: field emission scanning electron microscopy
FTIR: Fourier-transform infrared spectroscopy
MSB: minimal salt medium
CLSM: confocal laser scanning microscopy
FAME: fatty acid methyl esters
EDS: energy dispersive X-ray spectrometer
ANI: average nucleotide identity
