# Supplementary material for: Enhanced calcium carbonate-biofilm complex formation by alkali-generating Lysinibacillus boronitolerans YS11 and alkaliphilic Bacillus sp. AK13
Source: AMB Express. 2019 Apr 11;9:49. doi: 10.1186/s13568-019-0773-x (PMC6459448; doi:10.1186/s13568-019-0773-x)

AMB Express

Enhanced calcium carbonate-biofilm complex formation by alkali-generating *Lysinibacillus boronitolerans* YS11 and alkaliphilic *Bacillus* sp. AK13

Yun Suk Lee, Woojun Park*

Laboratory of Molecular Environmental Microbiology, Department of Environmental Science and Ecological Engineering, Korea University, Seoul, 02841, Republic of Korea

* Corresponding author: Dr. Woojun Park (wpark@korea.ac.kr)

Fig. S1. (A) Presence absence gene analysis of YS11 with other *Lysinibacillus* species including *L. boronitolerans* NBRC 103108^T, *L. macroides* DSM 54 ^{T}, *L. xylanilyticus* DSM 23493 ^{T}, and *L. pakistanensis* JCM 18776 ^{T}. (B) COG category of *Lysinibacillus* species.

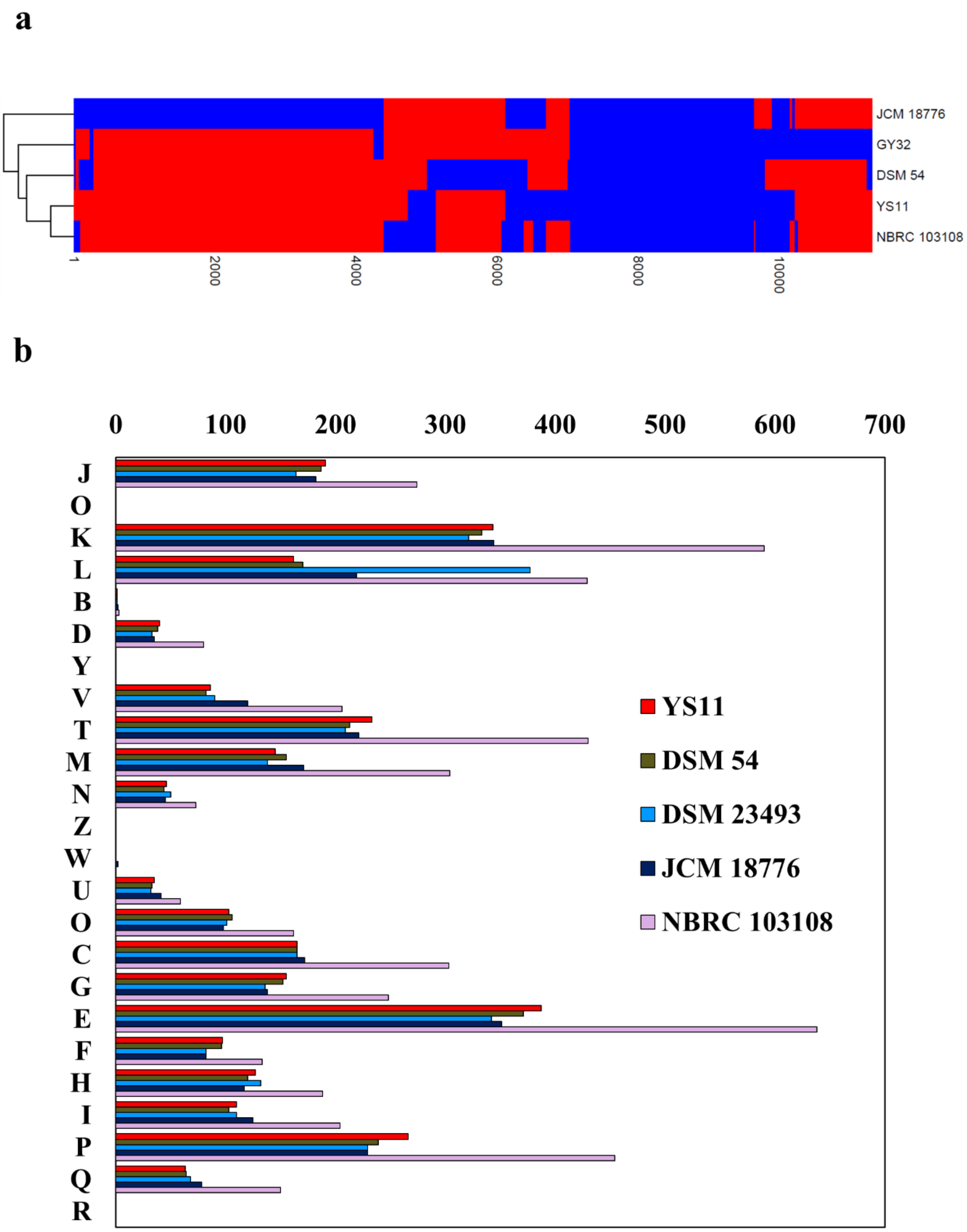

Fig. S2. (A) Phylogenetic neighbor joining tree of strain AK13. (B) pH dependent growth of alkalifying strain YS11 and alkaliphilic strain AK13.

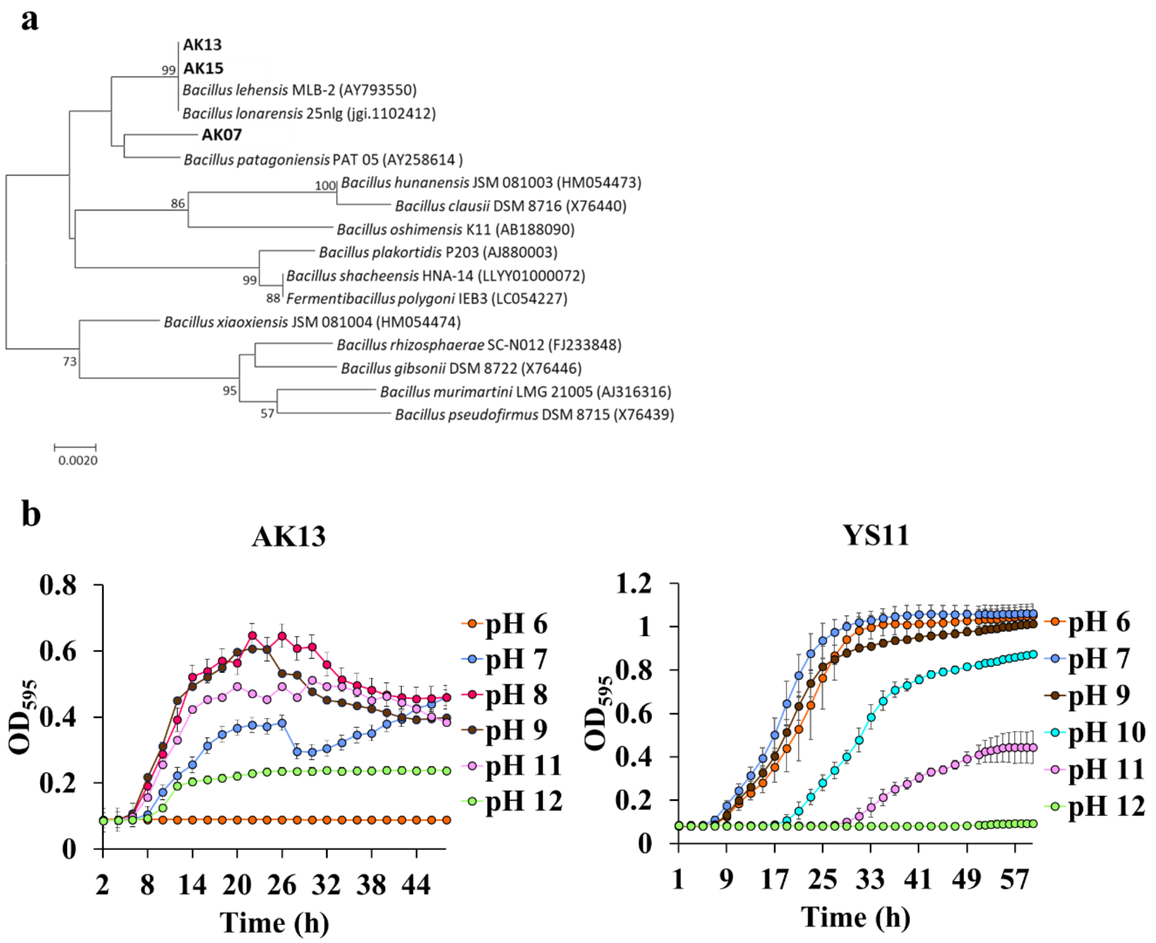

Fig. S3. FE-SEM and EDX analyses for nanoparticle calcium carbonate formed during early growth (6 h).

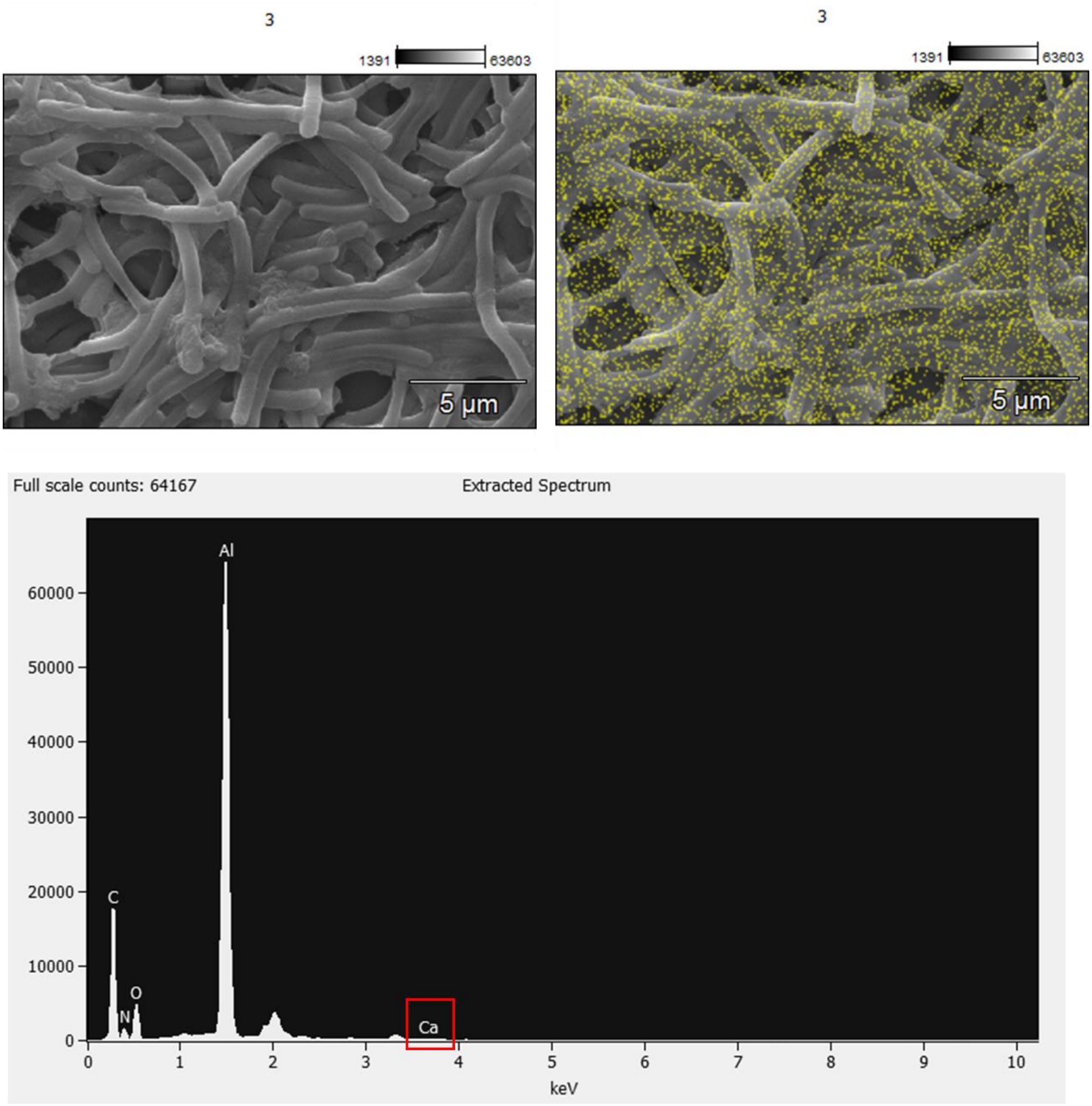

Supplement: Supplementary file 1 — Additional file 1: Fig. S1. (A) Presence absence gene analysis of YS11 with other Lysinibacillus species including L. boronitolerans NBRC 103108T, L. macroides DSM 54T, L. xylanilyticus DSM 23493T, and L. pakistanensis JCM 18776T. (B) COG category of Lysinibacillus species. Fig. S2. (A) Phylogenetic neighbor joining tree of strain AK13. (B) pH dependent growth of alkalifying strain YS11 and alkaliphilic strain AK13. Fig. S3. FE-SEM and EDX analyses for nanoparticle calcium carbonate formed during early growth (6 h). [file 13568_2019_773_MOESM1_ESM.pdf]
